# Effective and efficient active learning for deep learning-based tissue image analysis

**DOI:** 10.1093/bioinformatics/btad138

**Published:** 2023-03-21

**Authors:** André L S Meirelles, Tahsin Kurc, Jun Kong, Renato Ferreira, Joel Saltz, George Teodoro

**Affiliations:** Department of Computer Science, University of Brasília, Brasília 70910-900, Brazil; Biomedical Informatics Department, Stony Brook University, Stony Brook, NY 11794-8322, USA; Department of Mathematics and Statistics and Computer Science, Georgia State University, Atlanta, GA 30302-4110, USA; Department of Computer Science, Universidade Federal de Minas Gerais, Belo Horizonte 31270-901, Brazil; Biomedical Informatics Department, Stony Brook University, Stony Brook, NY 11794-8322, USA; Department of Computer Science, University of Brasília, Brasília 70910-900, Brazil; Biomedical Informatics Department, Stony Brook University, Stony Brook, NY 11794-8322, USA; Department of Computer Science, Universidade Federal de Minas Gerais, Belo Horizonte 31270-901, Brazil

## Abstract

**Motivation:**

Deep learning attained excellent results in digital pathology recently. A challenge with its use is that high quality, representative training datasets are required to build robust models. Data annotation in the domain is labor intensive and demands substantial time commitment from expert pathologists. Active learning (AL) is a strategy to minimize annotation. The goal is to select samples from the pool of unlabeled data for annotation that improves model accuracy. However, AL is a very compute demanding approach. The benefits for model learning may vary according to the strategy used, and it may be hard for a domain specialist to fine tune the solution without an integrated interface.

**Results:**

We developed a framework that includes a friendly user interface along with run-time optimizations to reduce annotation and execution time in AL in digital pathology. Our solution implements several AL strategies along with our diversity-aware data acquisition (DADA) acquisition function, which enforces data diversity to improve the prediction performance of a model. In this work, we employed a model simplification strategy [Network Auto-Reduction (NAR)] that significantly improves AL execution time when coupled with DADA. NAR produces less compute demanding models, which replace the target models during the AL process to reduce processing demands. An evaluation with a tumor-infiltrating lymphocytes classification application shows that: (i) DADA attains superior performance compared to state-of-the-art AL strategies for different convolutional neural networks (CNNs), (ii) NAR improves the AL execution time by up to 4.3×, and (iii) target models trained with patches/data selected by the NAR reduced versions achieve similar or superior classification quality to using target CNNs for data selection.

**Availability and implementation:**

Source code: https://github.com/alsmeirelles/DADA.

## 1 Introduction

Whole slide tissue specimens are routinely used in medical practice to diagnose cancer, determine cancer stage, and evaluate a patient’s response to treatment. Digital microscopy technologies make it feasible to rapidly and reliably digitize glass slides into Gigapixel images. Imaging features derived from whole slide images (WSIs) provide a quantitative tissue-based view of disease, which can improve disease diagnosis, disease classification, and prediction of clinical outcomes, as demonstrated by several studies (Angell and Galon 2013; Rakaee et al. 2018). Deep learning methods have recently shown high accuracy in several applications involving tissue image data (Barker et al. 2016; Saltz et al. 2018; Hossain and Sakib 2020).

Deep learning methods typically require a significant amount of labeled data for model training. This has motivated the development of a number of approaches to create large, representative training samples, and to train robust and accurate models with limited training data. Several projects have developed techniques to generate synthetic training datasets, consisting of pairs of synthetic tissue images and masks, for image segmentation (Hossain and Sakib 2020; Gong et al. 2021). Crowdsourcing approaches also have been implemented to curate large training datasets (Li and Deng 2018; Ørting et al. 2019).

Another strategy is an iterative model training process, called active learning (AL). This process starts with a small initial training dataset, which is iteratively augmented with new samples until a model with the desired accuracy has been trained. AL can be used in tandem with synthetic data generation and crowdsourcing; for example, a researcher may start with synthetic or crowd-sourced data and iteratively update the training data and refine the deep learning model by adding new samples. AL approaches with convolutional neural networks (CNNs) have already been shown success in various image analysis tasks (Gal et al. 2017; Sener and Savarese 2018).

A main task in AL is to collect samples that improve the accuracy of the model while minimizing the total number of samples (hence, the annotation cost) and the overall AL execution time. Sample selection strategies in most AL approaches are based on uncertainty metrics (Gal et al. 2017; Beluch et al. 2018) or on hybrid schemes that combine uncertainty with other metrics (Zhu et al. 2009; Li and Guo 2013; Yang et al. 2015; Yuan et al. 2019). In all cases, the metrics are computed against a pool of data samples that have not yet been labeled and are candidates to be included in the training dataset. The number of samples to be labeled can be large, making sample selection a very compute expensive step, increasing the execution time of the AL process.

In our previous work (Meirelles et al. 2022b), we approached this problem with the introduction of a diversity-aware sample selection method, called *DADA*, coupled with a sub-pooling strategy. We integrated it with the well-known Monte-Carlo (MC) dropout (Gal et al. 2017) and ensemble (ENS)-based AL methods (Beluch et al. 2018). DADA advances state-of-the-art in AL (notably, the MC and ENS approaches) by reducing the training dataset size required to attain a given model quality. Moreover, with sub-pooling, only a portion of the unlabeled data is evaluated in each AL iteration. DADA, nevertheless, has limitations which we address in this work. The execution time of the AL can still be very high with DADA. When the target deep learning architecture is complex, the AL process can require high-end computing capabilities to finish in a feasible time window. In addition, the computational cost can lead to long AL iterations—between the time the user annotates a set of images and the time a new set of images are presented to the user for annotation. This limits the interactive nature of AL and may lead to additional delays as the difficulty of synchronizing the user’s availability and the end of an AL iteration.

In this work, to address this limitation, we incorporate a method, called network auto-reduction (NAR), in the AL process. NAR is an approach for simplification of CNNs we developed (Meirelles et al. 2022a). The AL process with NAR reduces overall AL execution time by using simplified (hence, less computationally expensive) versions of the target networks in the sample selection step. The simplified CNNs can reduce both the training time required to generate the intermediary models in AL and also the prediction time over the pool of data, during uncertainty calculation. The experiments using our tumor-infiltrating lymphocyte (TIL) classification application show that the proposed method reduce the overall AL iteration time significantly. For instance, our NAR approach leads to an improvement of 2.6× and 4.3× for the MC DADA and ENS DADA, respectively, without reducing the overall quality of the model classification. In addition, performance gains grow as the training set increases. In the final iterations of the AL acquisition with ENS DADA, for instance, we were able to reduce execution times from close to 2 h to about 15 min (a speedup of about 8×). DADA was developed using typical deep learning tools that require interaction with system terminals that are not necessarily user-friendly interfaces for a domain expert, which can lead to limited adoption of AL in practice. To address this issue, we integrated our AL methods with a web-based interface, which supports annotation of tissue image patches, as an extension of the QuickAnnotator framework (Miao et al. 2021). This interface can enable a domain scientist to quickly employ and evaluate AL. The improvements in AL execution times by NAR benefit the use of this interactive tool because the user can annotate the patches quickly as the time between AL iterations are greatly reduced.

## 2 Materials and methods

**Figure 1:**
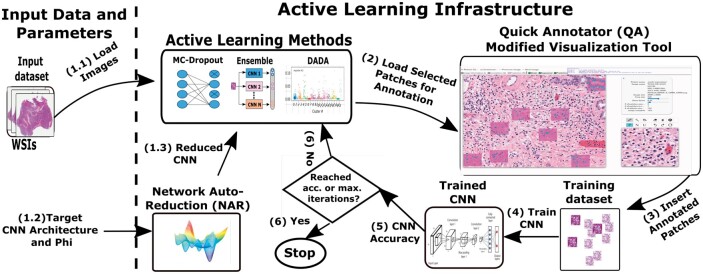
AL system architecture overview. The AL cycle starts by having the input data, model, and NAR parameters selected. The data are loaded and the model simplified by NAR. The AL methods select the patches to be annotated, which are then included in the training data. The CNN is then trained and reused to select new patches when the stopping criteria is not reached

The proposed AL methods, optimizations, and interfaces implemented in an unified framework are summarized in Fig. 1. Here we describe the main steps involved in an AL study and challenges addressed.

In order to carry out an AL study, a user (e.g. a pathologist) selects the WSIs of interest and a model architecture (Steps 1.1 and 1.2 in the figure). The images are partitioned into a pool of unlabeled patches. The target model is simplified using NAR (Step 1.3)—the current version of our software supports InceptionV4, ResNet50V2, and Xception out of the box. The first set of patches for annotation is chosen randomly (Step 2) from the pool; subsequent patch selections are done based on the classification results from the updated model in each AL iteration. The selected patches are shown in the context of the region in which they appear in the WSIs. The user assigns their classification labels. The target model is trained (Step 4) and may be evaluated to measure its classification quality, e.g. using the area under the curve (AUC) metric (Step 5). The AL termination condition is a user choice between classification quality and/or the maximum number of selected patches (Step 6). If the termination condition has not been evaluated true, the AL sample acquisition step is re-executed to select a new subset of patches for annotation by the user.

The process of selecting new patches to be annotated is complex both (i) in terms of choosing a method that is appropriate in the context of pathology image analysis and (ii) with respect to the computation time and computing power required. We have noticed, as will be presented in the experimental evaluation, that traditional AL methods published in the literature in other application domains (Gal et al. 2017; Beluch et al. 2018) do not perform well in digital pathology. First, there can be large areas with similar characteristics in a tissue specimen. Patches selected from these areas will likely have very similar visual characteristics. We observed that after a set of patches are processed by the target model and ranked based on prediction uncertainty, patches in the top of the ranked list (i.e. patches with the highest uncertainty) look alike and have very similar tissue characteristics. When patches with the highest prediction uncertainty are added to the training dataset, their contribution to the learning process will be small but they will add to the training cost. Second, the computation cost to decide which patches should be included in the training dataset can be high because of the large number of patches in the unlabeled pool, for which prediction uncertainty values are to be computed, as well as because of the complex computations required by the existing AL methods (Gal et al. 2017; Beluch et al. 2018). DADA and NAR used in tandem address these challenges. The remaining of this section presents our motivating application, reviews AL methods from previous work in the literature, and describes the proposed optimizations.

### 2.1 Target application

Patch-based prediction and classification of spatial patterns of imaging features in WSIs is a common analysis method in digital pathology. In this method, a WSI is partitioned into a uniform grid of patches. A deep learning model is trained to assign one or more classes to each patch. In this work, we use an application that predicts and classifies distributions of TIL in WSIs in our experimental evaluation of the proposed AL methods and optimizations. Recent studies have shown that a consistent correlation exists among TIL patterns in a tissue and important aspects as cancer progression and patient outcome (Mlecnik et al. 2011; Angell and Galon 2013; Berghoff et al. 2016; Rakaee et al. 2018). In previous works (Saltz et al. 2018; Le et al. 2020), we developed deep learning methods that predict distributions of TILs in WSIs and examine their spatial patterns in several cancer types.

**Figure 2:**
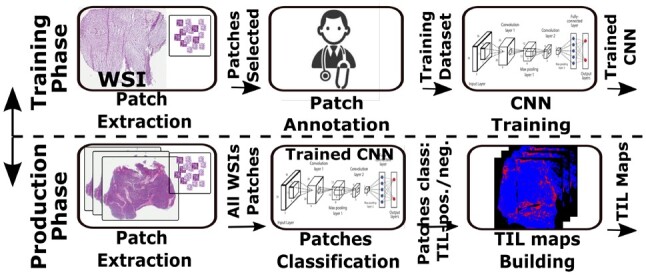
TIL application training and production workflows. Input WSIs are partitioned into patches that receive a TIL rich positive/negative classification. The output is a heatmap showing the classification. (a) Comparison of AL strategies. (b) Execution times of AL strategies

In these methods, a WSI is uniformly partitioned into image patches of 100 × 100 or 200 × 200 square pixels (Fig. 2). An annotator selects regions of interest and annotates the patches in the selected regions. This region selection and patch annotation process repeats until a reasonable set of labeled patches is created. The set of patches is used to train a classification model, which predicts if an unseen patch is TIL positive, i.e. if the patch contains lymphocyte cells.

In an AL setting, the region selection is not necessary. Instead, patches to be annotated are selected by the AL process and presented to the user. A whole slide image, however, can contain hundreds of thousands of patches. Hence, selecting the best set of patches for annotation in each AL iteration can be a challenging and expensive task.

### 2.2 AL methods

AL is an iterative process consisting of model training, model inference, dynamic selection of unlabeled data points (also called data acquisition), annotation of the selected data points, and model refinement or re-training steps. The data acquisition step selects a set *A* of data points/items to be annotated from a typically large pool of unlabeled data *U* using an acquisition function. At iteration *i* of an AL process, the current machine learning model $M^{i}$, built with training set $L^{i}$, is applied to $U^{i}$, and a *selection metric* (*uncertainty estimates being the most common*) is computed for each data point in $U^{i}$. The acquisition function $a(U^{i},M^{i})$ selects the best data points according to the metric to create the set of $A^{i+1}$ samples to be annotated for the next iteration of the AL process (Fu et al. 2013). The pool and training sets are updated at each iteration as follows: $L^{i+1}=A^{i}\cup L^{i}$ and $U^{i+1}=U^{i}-A^{i}$. This process is executed until a stopping condition is satisfied, e.g. the size of $L^{i}$ or the model accuracy exceeds a threshold.

Uncertainty computation with CNNs requires a probabilistic approach. Two popular strategies are Monte-Carlo (MC) Dropout (Gal et al. 2017) and Ensembles (ENS) (Beluch et al. 2018). MC uses dropout to approximate a CNN model posterior, meaning that dropout regularization is seen as a variational Bayesian approximation. By using dropout in training and testing (stochastic forward pass), prediction variance is computed from results of *F* passes with different dropout masks. ENS computes uncertainty by using *N* CNN models built with different initialization weights. Classifications from those models lead to variations.

Multiple uncertainty functions used in other domains have been adapted to CNNs (Gal et al. 2017) and are used with MC and ENS. The most common are (i) Max Entropy, which measures the amount of information that can be extracted from a process, (ii) BALD (Houlsby et al. 2011), which chooses items that maximize the mutual information of prediction and model posterior, or those producing disagreeing predictions with high certainty, and (iii) variation ratios (VR) (Freeman and Freeman 1965), which selects items with most dispersed classification labels.

DADA is an acquisition function we proposed in earlier work (Meirelles et al. 2022b). It is motivated by our observation that the previous AL strategies tend to select similar items, limiting the contributions of the newly selected data items to model improvement (refer to Supplementary Section S3). DADA modifies the acquisition process with a data grouping (clustering) based on feature information to enforce diversity. This is implemented by combining data grouping and uncertainty, such that data items selected from each group are those with the highest uncertainty.

DADA first computes deep features from the model being trained to describe the unlabeled data items. A reduction step is executed to restrict data dimensionality with the principal component analysis (PCA). The data items are then clustered into groups based on the distance between items in the feature space. Data items in each group are sorted with respect to their uncertainty values (computed using MC or ENS functions). A set of patches (*acqSize*) with the highest uncertainty is acquired from each group. If Z(i) is the average uncertainty of cluster *i* and $PR(i)=Z(i)/\sum_{K}Z(i)$, *acqSize* is equal to PR(i)*Q, given a patch acquisition target per iteration *Q* and *K* clusters.

In essence, *acqSize* is weighted by the average uncertainty of data items in the groups. Because the unlabeled pools of data items can be very large, DADA implements a subpooling strategy in which only a portion of the pool is considered in each AL iteration. The subpool is created by sampling the original pool ($U^{i}$) and by replacing it in each AL iteration. The number of clusters is determined experimentally, but as presented in Supplementary Section S3, the AUC values attained in the AL were relatively stable with respect to this parameter. Cluster numbers from 10 to 80 obtained good performance.

### 2.3 Accelerating AL with reduced CNNs

DADA can improve a model’s classification accuracy quickly, hence reduce the overall annotation effort. Nevertheless, each AL iteration may take a long time depending on the pool size, training dataset, and the complexity of the target CNN architecture.

We propose a strategy that replaces the target CNN with a less resource demanding or compact CNN to be used in the AL loop. Our approach automatically creates a simplified CNN via a process referred to as *Network Auto-reduction* (*NAR*) (Meirelles et al. 2022a). NAR simplifies the target CNN in a systematic manner by reducing the number of layers, channels, and tensor resolutions. In this way, the reduced CNN will be less compute demanding but will preserve the properties of the original CNN.

Common techniques for CNN simplification focus on reducing one of the network dimensions: depth, width, or input resolution. Other works also reduce CNN parameters either by eliminating specific filters (Cheng et al. 2017; Luo et al. 2018; Ding et al. 2019; Osaku et al. 2021), introducing weight sparsity (Shao et al. 2021) or a combination of both (Lin et al., 2019; Ding et al. 2021). Most simplification strategies require the CNN to be executed in a sequence of retraining steps to decide specific parts to be removed. This is rather costly and not viable in AL.

NAR is built on the concepts and methods developed by Tan and Le (2019) to scale up simple CNNs. Their objective was to improve model performance while keeping the complexity of the up-scaled CNN in check. Here, we use their approach to move in the opposite direction by reducing all the dimensions of a given CNN. Our goal is to reduce the computational complexity of the CNN while maintaining good classification performance. The scaling objective is formulated in (Tan and Le 2019) as an optimization problem as follows: where $\underset{i=1,\ldots ,s}{⊙}$ represents the concatenation of layers ${\hat{L}}_{i}$ that make the model M. Each layer ${\hat{L}}_{i}$ can be viewed as the application of function ${\hat{F}}_{i}$ on input tensor *X*, with dimensions ${\hat{H}}_{i},{\hat{W}}_{i},{\hat{C}}_{i}$ (height, width, and channels). Every layer ${\hat{L}}_{i}$ can be repeated in a sequence of occurrences forming blocks. The scaling process increases all three dimensions of a network simultaneously, *depth* (the number of layers ${\hat{L}}_{i}$), *width* (the number of channels ${\hat{C}}_{i}$), and *resolution* (the height ${\hat{H}}_{i}$ and width ${\hat{W}}_{i}$ of tensor *X*) in a balanced way. The scaling coefficients *d*, *w*, *r*, enable creating an enlarged model M with $d.{\hat{L}}_{i}$ occurrences of layer *i* and input size $r.{\hat{H}}_{i},r.{\hat{W}}_{i},w.{\hat{C}}_{i}$, except for layer i=0 in which the number of channels correspond to the input image channels.


(1) $$\;\begin{array}{c} \underset{d,w,r}{\max}\;\;\mathrm{Accuracy}(M(d,w,r))\\ s.t\;\;M(d,w,r)=\underset{i=1,\ldots ,s}{⊙}{\hat{F}}_{i}^{d.{\hat{L}}_{i}}\left(X_{\langle r.{\hat{H}}_{i},r.{\hat{W}}_{i},w.{\hat{C}}_{i}\rangle }\right) \end{array},$$

The increase in the floating point operations (FLOPS) cost is proportional to $d.w^{2}.r^{2}$. To balance the distribution of the cost among these parameters, a ϕ coefficient is used, such that $d=\alpha^{\phi },w=\beta^{\phi },\text{and}\;r=\gamma^{\phi }$. Tan et al. chose to apply a restriction to the hyperparameters as follows: $\alpha .\beta^{2}.\gamma^{2}\;\approx 2$. α,β, and γ are defined by a model grid search. Given these conditions, for every integer value of ϕ, FLOPS cost would be increased by $2^{\phi }$, whereas depth, width, and resolution would be increased by an uniform amount. The restrictions together with the ϕ parameter create an easy-to-evaluate FLOPS target for the enhanced network.

In our approach, we employ a similar strategy, where a reduction factor is applied to each dimension, given by: $d=\alpha^{-\phi },w=\beta^{-\phi },\text{and}\;r=\gamma^{-\phi }$ with the same restrictions valid for α, β, and γ. This, in turn, results in a theoretical FLOPS reduction of $\frac{1}{2^{\phi }}$ for every value of ϕ. Therefore, NAR creates reduced versions of any block-based CNN using a single user defined parameter ϕ, which allows for a trade-off between computational cost and model classification performance. The ϕ parameter enables the user to estimate network simplification as a matter of FLOPS. In this way, simplified CNNs can be generated that would execute in a feasible time on available hardware. While a simplified CNN may affect the quality of patches selected during the AL process, we observed in our experiments that ϕ values of up to 5 have negligible impact on the quality of the models (see Table 1). Sequential models, such as VGG (Simonyan and Zisserman 2014), can also be reduced with the use of a binary selection that removes or keeps each convolutional layer. The configurations of the CNNs used in this study after reduction are shown in Supplementary Tables S5–S7.

**Table 1.** Mean AUC levels with NAR and data augmentation.^a^

Approach	Original						Data augmentation					
	DADA	ϕ=2	ϕ=3	ϕ=4	ϕ=5	ϕ=6	DADA	ϕ=2	ϕ=3	ϕ=4	ϕ=5	ϕ=6
MC dropout	0.76	0.72	0.76	0.73	0.69	0.72	0.80	0.83	0.82	0.81	0.82	0.81
Ensembles	0.76	0.79	0.75	0.70	0.62	0.66	0.80	0.83	0.83	0.83	0.84	0.82

a Inception V4 is the target CNN.

Although the best values for α, β, and γ are model specific, Tan et al. used the values obtained for EfficientNet to well established networks, as ResNet-50 (He et al. 2016) and MobileNet (Sandler et al. 2018), with good results (Tan and Le 2019). In our pruning strategy, the same values are used: α=1.2, β=1.1, and γ=1.15.

## 3 Results

We evaluated the proposed method with the application described in Section 2.1 and referred to here as the TIL application and with three CNN architectures (InceptionV4, ResNet50V2, and Xception). We used a dataset of image patches generated from a set of 62 WSIs from 10 cancer types, including breast, prostate and pancreatic cancer cases (Supplementary Tables S1–S4) in The Cancer Genome Atlas (TCGA) repository. Each image patch covered a tissue area of 50 μm × 50 μm and was resized to 240 × 240 pixels. The dataset was unbalanced with only 16% of TIL positive patches. We used 57 WSIs to create the training and validation sets and the remaining 5 WSIs for testing. The dataset details are presented in Supplementary Section S1.

The number of patches in the validation and test sets was set to 100 and 15 000, respectively. The number of patches in the initial unlabeled pool was 100 000. The MC Dropout and Ensemble methods selected patches from this pool. DADA, on the other hand, used subpools of 2000 patches—the subpools were dynamically created from the entire pool during AL. The MC uncertainty calculation method executed F = 20 forward passes; the Ensemble method employed *N* = 3 models. In all cases, the models were trained from scratch for 50 epochs using Adam optimizer and initial learning rate of $1\times 10^{-4}$.

The experiments were executed on a Linux machine with 2 20-core Intel Xeon Gold 6248 CPUs, 512 GB of DDR4 RAM, and an NVIDIA Tesla V100 GPU with 32 GB of dedicated memory. The codes were developed using Keras and Tensorflow. The results with confidence interval report the standard deviation of five executions.

### 3.1 Performance of DADA with full CNNs

This section presents a brief evaluation of the efficacy and performance of the AL strategies with the full Inception V4 architecture. The experiments compare MC and ENS with and without DADA. In the experiments, the BALD uncertainty function achieved higher area under the ROC curve (AUC) values than the Max Entropy and VR function; hence, the results presented in this section were obtained with BALD.

**Figure 3:**
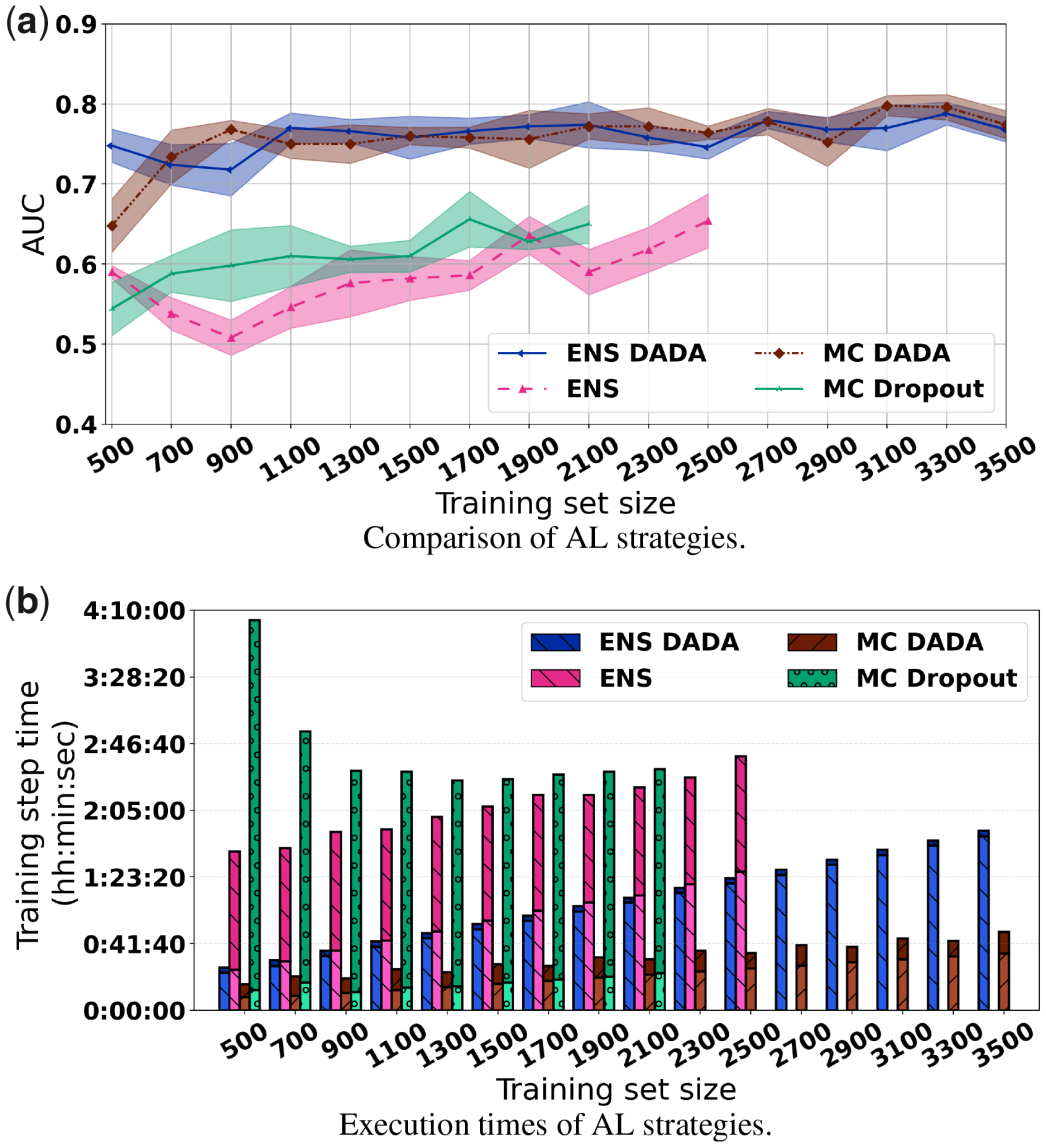
Comparison of the state-of-the-art AL strategies in the TIL application. The ENS/MC DADA approaches attained better AUC and smaller execution times versus ENS and MC Dropout without DADA. (a) Model performance with MC DADA and NAR for ϕ varied between 2 and 6. (b) Model performance with ENS DADA and NAR for ϕ varied between 2 and 6

Figure 3 shows that MC and ENS without DADA attain lower model performance and higher execution times as compared to MC and ENS with DADA (MC DADA and ENS DADA). This is because MC DADA and ENS DADA select patches with different characteristics from the pool of unlabeled data, promoting diversity in a controlled manner. If only uncertainty is considered, as in the original MC and ENS methods, very similar patches which have high uncertainty are added to the training data resulting in relatively small benefit to training. We additionally executed an experiment in which Inception V4 is trained with a large training set of 60 000 patches selected randomly from our dataset. A maximum AUC of 0.76 was achieved in the experiment. This again demonstrates the ability of our AL methods to reduce annotation requirements; out method, DADA, attained a similar AUC with only 2700 patches.

The AL processes with MC and ENS without DADA took much longer to execute than those with MC DADA and ENS DADA as shown in Fig. 3b—MC and ENS timed out after 2100 and 2500 patches had been selected because of the 30 h execution time limit in the job queues on the machine used in the experiments. The shorter execution times of MC DADA and ENS DADA are a result of subpooling used by DADA, which reduces the number of uncertainty computations to a small subset of the patches in the entire pool. The MC strategy had higher execution times in the earlier iterations of the AL process. Since uncertainty calculation in MC requires 20 forward passes over the pool (100k patches), all the patches had to be staged in memory to speedup computations (45 GB of RAM were needed). After the patches were loaded, execution times stabilized for the reminder of the experiment.

MC DADA attained a slightly higher AUC mean value and was close to 2× faster than ENS DADA. Therefore, we used MC DADA in the experiments described in the next sections. We also show results with ENS DADA in the next section to demonstrate that our optimizations work with the ENS approach as well.

### 3.2 Impact of NAR to quality and performance

While DADA can reduce the AL execution time significantly as demonstrated in the previous section, each iteration of the AL loop may still take tens of minutes. In the experiments in this section, we applied NAR to simplify the Inception V4 network by varying ϕ (depicted as PHI in the figures) between 2 and 6.

**Figure 4:**
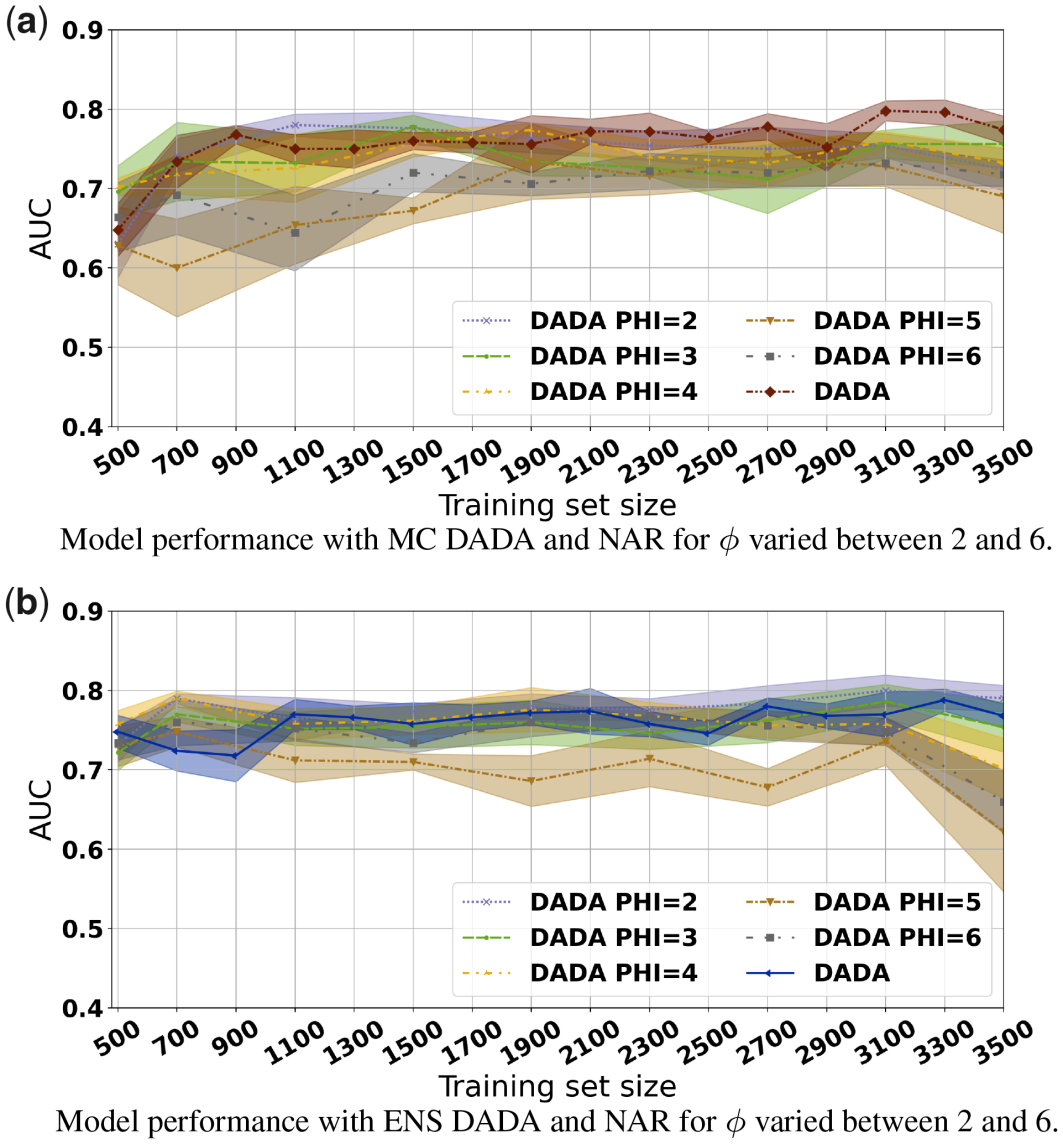
Impact of NAR on (a) MC DADA and (b) ENS DADA with different ϕ values. Inception V4 simplified by NAR has competitive AUC versus the original Inception V4. (a) Execution time per acquisition for MC DADA for multiple ϕ values. (b) Execution time per acquisition for ENS DADA for multiple ϕ values

**Figure 5:**
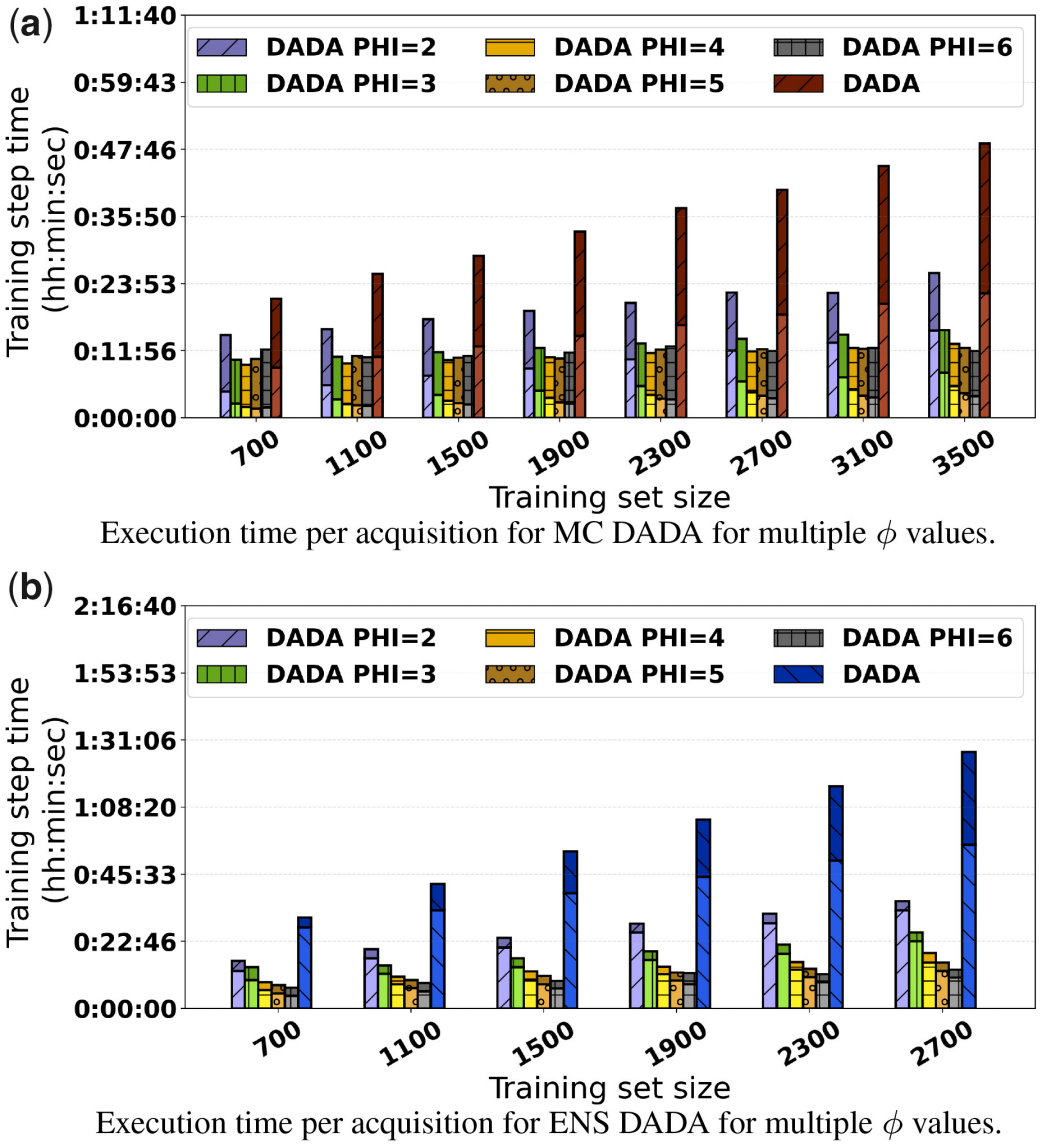
Execution time of NAR varying ϕ for MC/ENS with InceptionV4. Significant runtime reductions are observed with the use of NAR

The AUC values are presented in Fig. 4 for MC DADA and ENS DADA. The values were attained by the full Inception V4 trained with patches selected by the reduced Inception V4 in the AL loop. As is shown in Fig. 4a, the AUC values with patches selected with ϕ equal to 2, 3, and 4 are very competitive, or within <1 SD, as compared to the case in which the full network is used in the AL loop (MC DADA). The use of the reduced network in the AL has resulted in significant reduction in execution times (Fig. 5). For instance, MC DADA ϕ=3 attains an AUC of 0.78 while MC DADA (original CNN) reaches 0.80, whereas the first is 2.5× faster.

We also note that if the reduced network were trained and evaluated in the test phase, its classification results (AUC) would be lower than using the full network. For instance, for patches selected using the reduced network with ϕ values 2, 4, and 6, the maximum AUC of the full network would be 0.78, 0.77, and 0.73, whereas the reduced network with the same ϕ values would attain AUC values of at most 0.76, 0,72, and 0.71. This demonstrates that while NAR results in CNNs that are effective in selection of good data items in the AL process, their own classification quality may degrade with the network reduction.

A similar pattern is observed in another analysis carried out with the ENS strategy as is shown in Fig. 4b. The AUC values with ϕ of up to 4 are competitive to those when the full network (ENS DADA) is used in the AL loop. When ϕ=4, the execution time of the AL loop is 4.3× faster than that with ENS DADA (Fig. 5b).

### 3.3 Impact of NAR with other CNN architectures

This section evaluates NAR with Xception and ResNet50. Supplementary Figure S4a shows the AUC values of Xception, ResNet50, and Inception V4 with MC DADA. There is a significant difference in the AUC values attained by the CNNs for the same training set size, with Inception V4 being the best choice, followed by Xception and ResNet50. The overall AL execution times for Inception V4, Xception, and ResNet50 are presented in Supplementary Fig. S4b. As shown, Inception is only about 1.27× slower than Xception.

The results of the experiments with NAR are presented in Supplementary Figs S5 and S6. Note that ResNet50 is not as deep as the other architectures. Hence, a ϕ value greater than 4 is not possible given the block structure of the model, which limits the possible impact of NAR. As is shown in the figures, the quality of the selected patches and the AUC values degrade as the value of ϕ is increased, as expected. However, the AUC difference between using the full CNN and the reduced CNN with ϕ=2 is minimum. For instance, the difference is 3% and 2.5% for Xception and ResNet50, respectively, while an execution time reduction of over 2× is observed.

### 3.4 Cross-architecture patch selection

Another approach for reducing the execution time of the AL loop is to employ a full CNN that is smaller and less computation intensive than the target model in the AL loop. In this set of experiments, we used Xception and ResNet50, which are faster than Inception V4, in the AL loop as the *reduced* networks to select patches. We compared this strategy to the NAR approach with ϕ=3.

The mean AUC values across all iterations were 0.69 and 0.72 when Xception and ResNet50 chose patches, whereas NAR (ϕ=3) achieved an AUC value of 0.76. The maximum AUC values when using Xception, ResNet50, and NAR ϕ=3 with MC DADA were 0.74, 0.75, and 0.78, respectively. Inception V4 simplified by NAR has attained higher maximum and mean AUC values than the other solutions in all cases. A reason for the better quality may be that as NAR reduces the target CNN, it preserves the overall structure of the network. Consequently, more appropriate patches may be selected for that architecture. MC DADA and NAR with PHI = 3 was 1.92× and 2.14× faster than when Xception and ResNet were used in patch selection, respectively.

### 3.5 Training with data augmentation

This section presents the performance of Inception V4 when it is trained with patches selected by the previous approaches and with data augmentation (DA). Tissue staining can vary across tissue samples, resulting in WSIs with variations in color and intensity values. To minimize such variations, we normalized the RGB channels with a target mean and standard deviation of 0.0 and 1.0, respectively. We then augmented the training dataset by performing data rotations (of up to 22.5°), random vertical and horizontal flipping, and perturbations in patch brightness, contrast, and saturation. This is only performed in the training dataset, which consists of the 3500 patches selected by the AL.

Table 1 presents the mean AUC values of five experiments, using Inception V4 trained with patches selected by the original and reduced versions of the network in the MC DADA and ENS DADA approaches. DA significantly improved the AUC values of the models as expected, leading to high AUC values of up to 0.87 in some experiments with MC DADA. Moreover, the quality of the models with patches selected by the reduced network was comparable or superior to those using the original network. The results demonstrate again that our approach can reduce AL execution times with minimal impact, and in some cases with improvements, to the performance of the trained models.

## 4 Discussion

Recent advances in deep learning (DL) methods are increasingly benefiting digital pathology. However, there still are challenging issues to be addressed in order to make DL even more useful in the domain. One major challenge is the limited availability of annotated datasets required to train DL models (Amgad et al. 2019; Sakamoto et al. 2020; Morales et al. 2021; van der Laak et al. 2021; Wang et al. 2021). This is specially important in digital pathology because annotating images for training data is a labor-intensive task and demands input from expert pathologists.

We propose and evaluate the use of AL in digital pathology to reduce the data annotation effort. We approach the problem with an end-to-end solution that is deployed into a visualization tool that includes a user-friendly interface for data annotation, an effective AL strategy for pathology image analysis, and run-time optimizations to speedup the AL execution, which enables interactive and quicker training data generation.

While AL is demonstrated to be effective in image analysis for several domains, state-of-the-art AL techniques widely used in other applications may not improve model quality significantly for a given annotation dataset size in digital pathology (Carse and McKenna 2019; Rączkowski et al. 2019). This has motivated a new approach (DADA) that reduces the number of patches annotated to attain the desired accuracy. DADA improves other strategies by enforcing data diversification in the AL selection while selecting the most uncertain patches with each visual (cluster)-based patch group. We have also assessed the diversification through a visual examination and a spatial analysis of selected patches. The visual evaluation (Supplementary Fig. S2) shows a significant heterogeneity on the tissue in patches of different clusters when using ENS DADA. The most uncertain patches when using ENS without DADA are also presented in Supplementary Fig. S3, which illustrates that very similar patches are selected. The spatial analysis demonstrates that patches selected from the same group come from a significant number of different slides. Those in the same slide are typically far from each other (as measured by the distance in number of pixels—Supplementary Table S8).

AL requires significant computing power to compute data selection. This, in turn, could lead to multiple annotation cycles (AL steps) with a temporal gap among them in the order tens of minutes or close to an hour. This is another aspect that reduces the efficiency of the annotation process, as the user will have switch between tasks (annotation and another work) to not sit idle until the next set of patches is ready to be labeled. In order to reduce the time to select new patches, we have proposed the use of reduced and less computationally demanding versions of the target model that are automatically generated by the NAR method. Our experimental results show that this leads to a reduction in the execution time in the order of 4×, reducing the gap between the end of one AL iteration and when patches are availability for annotation to about 10 min. We show that this strategy works for different CNN architectures and that a reduced CNN select more appropriate patches than when a different architecture is used.

Our approaches have improved AUC levels and speed of AL in the TIL application. The deployment of the solutions in an easy to use interface will simplify its use in other applications. Consequently, we are currently evaluating the use of AL with other applications in the digital pathology domain. This study is being carried out with pathologists directly interacting with the annotation and AL framework presented in this article. This will enable us to expand our analysis with a qualitative evaluation of the data selected by our AL methods.

## Supplementary Material

btad138_Supplementary_Data
