# Supplementary material for: Effective and efficient active learning for deep learning-based tissue image analysis
Source: Bioinformatics. 2023 Mar 21;39(4):btad138. doi: 10.1093/bioinformatics/btad138 (PMC10079352; doi:10.1093/bioinformatics/btad138)
Supplement: btad138_Supplementary_Data [file btad138_supplementary_data.pdf]

Supplementary Materials for Effective and Efficient Active Learning for Deep Learning Based Tissue Image Analysis

André L. S. Meirelles[1], Tahsin Kurc[2], Jun Kong[3], Renato Ferreira[4], Joel Saltz[2], and George Teodoro[1,2,4,*]

[1]Department of Computer Science, University of Brasília, Brazil.
[2]Biomedical Informatics Department, Stony Brook University, USA.
[3]Department of Mathematics and Statistics and Computer Science, Georgia State University, Atlanta, GA, USA.
[4]Department of Computer Science, Universidade Federal de Minas Gerais, Belo Horizonte, 31270-901, Brazil.
[*]To whom correspondence should be addressed. Tel +55-61-3107-3663; Email: george@dcc.ufmg.br.

March 15, 2023

S1 Imaging Data Used

All cancer WSI images used in our evaluation were collected from The Cancer Genome Atlas (TCGA) (National Human Genome Research Institute, 2017) repository. The number of slides and types of tissues used in our experiments are given by Tables S1 and S2.

Acronym	Tumor type
BLCA	Bladder urothelial carcinoma
BRCA	Breast invasive carcinoma
CESC	Cervical squamous cell carcinoma and endocervical adenocarcinoma
COAD	Colon adenocarcinoma
PAAD	Pancreatic adenocarcinoma
PRAD	Prostate adenocarcinoma
READ	Rectum adenocarcinoma
SKCM	Skin Cutaneous Melanoma
STAD	Stomach adenocarcinoma
UCEC	Uterine Corpus Endometrial Carcinoma

Table S1: Tumor types.

	PRAD	UCEC	BLCA	STAD	SKCM	COAD	BRCA	CESC	PAAD	READ
Train/Val	5	13	3	9	8	4	9	5	0	1
Test	0	0	1	0	2	0	0	1	1	0
Total	5	13	4	9	10	4	9	6	1	1

Table S2: Tissue types and number of slides for each.

Slide TCGA ID that are part of the training/validation set are presented in Table S3. The test set is formed by slides of TCGA ID given by Table S4.

TCGA-EO-A22S-01Z-00	TCGA-FI-A2EY-01Z-00	TCGA-S5-AA26-01Z-00	TCGA-C5-A7CK-01Z-00	TCGA-EY-A3L3-01Z-00
TCGA-HG-A9SC-01Z-00	TCGA-EY-A2OO-01Z-00	TCGA-BS-A0UF-01Z-00	TCGA-BH-A202-01Z-00	TCGA-D7-6519-01Z-00
TCGA-AP-A0LH-01Z-00	TCGA-OD-A75X-06Z-00	TCGA-BG-A0W1-01Z-00	TCGA-BR-6709-01Z-00	TCGA-AP-A1DM-01Z-00
TCGA-BG-A0MQ-01Z-00	TCGA-W3-AA1V-01Z-00	TCGA-D3-A8GJ-06Z-00	TCGA-VQ-A8P8-01Z-00	TCGA-MY-A913-01Z-00
TCGA-XF-AAMX-01Z-00	TCGA-EE-A3AF-01Z-00	TCGA-D9-A149-01Z-00	TCGA-DS-A7WF-01Z-00	TCGA-EW-A1J1-01Z-00
TCGA-D1-A16N-01Z-00	TCGA-VQ-A94U-01Z-00	TCGA-VQ-A8DT-01Z-00	TCGA-EJ-5497-01Z-00	TCGA-BG-A0M4-01Z-00
TCGA-BR-8058-01Z-00	TCGA-C8-A12K-01Z-00	TCGA-D5-6534-01Z-00	TCGA-YL-A8HL-01Z-00	TCGA-EW-A1PF-01Z-00
TCGA-D1-A176-01Z-00	TCGA-F5-6864-01Z-00	TCGA-BR-8682-01Z-00	TCGA-HC-7078-01Z-00	TCGA-AM-5820-01Z-00
TCGA-EY-A549-01Z-00	TCGA-D8-A1XD-01Z-00	TCGA-XV-AAZW-01Z-00	TCGA-HC-8264-01Z-00	TCGA-EE-A2GS-01Z-00
TCGA-AU-6004-01Z-00	TCGA-FR-A7U8-01Z-00	TCGA-HU-A4GP-01Z-00	TCGA-HU-8610-01Z-00	TCGA-VS-A8EJ-01Z-00
TCGA-AO-A0JI-01Z-00	TCGA-EJ-5530-01Z-00	TCGA-E9-A3X8-01Z-00	TCGA-BH-A0BR-01Z-00	TCGA-AY-6196-01Z-00
TCGA-D8-A1XU-01Z-00	TCGA-C4-A0F6-01Z-00			

Table S3: TCGA slide IDs for the training/validation set.

TCGA-BL-A13J-01Z-00	TCGA-FR-A728-01Z-00	TCGA-EE-A2MH-01Z-00	TCGA-C5-A1MH-01Z-00	TCGA-US-A77G-01Z-00

Table S4: TCGA slide IDs for the test set.

The slides listed in Tables S3 and S4 were randomly selected from a total of 5,202 images used in Saltz *et al.* (2018). These slides were divided into patches, generating a total of 525,431 of them, of which 15,000 correspond to the test set and originates from WSIs in Table S4, 100 form a validation set and the remaining patches form the pool of unlabeled data.

The number of generated patches is very large, making it impractical to be used in an AL scheme. Thus, in the early stages of DADA (Meirelles *et al.* (2022)), a fixed pool of 100,000 randomly selected patches was created. This selection can be seen as an individual TIL dataset and different selections can be made from the overall patch set extracted.

S2 Parameters of CNNs Used with NAR

This section presents complementary information about the CNNs used during experiments and their reduced versions (Tables S5 to S7).

It is important to point out that since ResNet50 is a shallower network than Inception and its architecture has a block structure that would convert blocks into sequential layers if higher ϕ values were used, NAR was limited to $\phi = 3$ for this model.

CNN	# Params	Param. Layers	# of Layers	Input Dim.
Inception V4	54,385,570	245	861	240x240
$\phi = 2$	28,118,858	179	627	181x181
$\phi = 3$	20,002,127	145	507	158x158
$\phi = 4$	14,506,100	123	429	137x137
$\phi = 5$	10,275,579	101	351	119x119
$\phi = 6$	7,484,162	91	315	104x104

Table S5: Number of parameters and layers in the original Inception V4 (binary classification) and reduced networks.

CNN	# Params	Param. Layers	# of Layers	Input Dim.
ResNet50 V2	23,568,898	54	222	240x240
$\phi = 2$	11,274,413	39	157	181x181
$\phi = 3$	8,514,988	33	131	158x158

Table S6: Number of trainable parameters and layers in the original ResNet50 V2 (binary classification) and reduced networks. The numbers include shortcut connections.

S3 Cluster configuration

Defining the number of clusters to be used with DADA requires experimentation and/or knowledge about dataset characteristics. For the MNIST dataset, which consists of handwritten decimal digits, it is natural to assume that 10 clusters may be a good choice, although that does not necessarily correspond to the best configuration.

The TIL dataset does not have an obvious first choice for this parameter and experimentation was needed to point out a candidate. Figure S1 presents the results of these experiments. Average AUC for the 20, 40 and 80 clusters configurations was, respectively, 0.735, 0.739 and 0.732, showing minor differences, while 10 and 200 averaged at 0.724 and 0.697. Given these findings, the 20 cluster size was chosen, but other values with similar AUC could also be used.

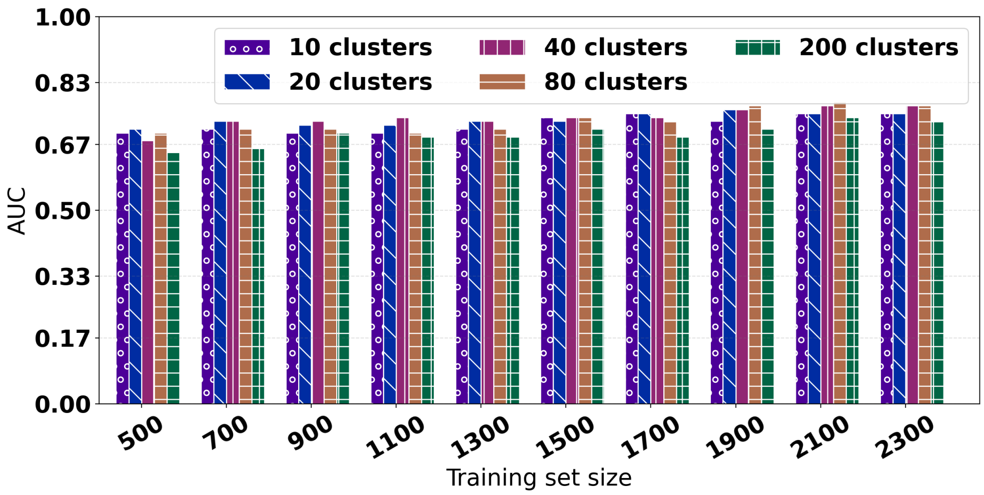

Figure S1: Number of clusters analysis for ENS DADA.

CNN	# Params	Param. Layers	# of Layers	Input Dim.
Xception	20,811,050	41	148	240x240
$\phi = 2$	12,049,631	35	126	181x181
$\phi = 3$	9,050,232	32	115	158x158
$\phi = 4$	6,729,329	29	104	137x137
$\phi = 5$	4,947,572	26	93	119x119
$\phi = 6$	4,096,797	26	93	104x104

Table S7: Number of trainable parameters and layers in the original Xception (binary classification) and reduced networks. The numbers include shortcut connections.

In order to illustrate the effect of DADA in patch selection, Figure S2 shows the 5 patches with highest uncertainty in DADA's 20 clusters, used during image selection. These patches come from iteration # 19 of an Ensemble DADA experiment, in which 20 iterations were carried out (the last iteration does not execute patch selection). In this iteration, a subpool of 1,800 patches was available to be chosen by DADA and they were distributed among the 20 patch clusters. The number of selected patches from each cluster depends on the cluster's average uncertainty.

Additionally, patches within clusters usually span multiple slides and even when a subset of them come from the same slide they are not necessarily spatially close to each other. This is a result of the subpooling/sampling approach, in which only a subset of the pool is available to the selection strategy at each iteration, with subpool regeneration carried out after every α acquisitions.

Table S8 shows the maximum and average distance between pairs of patches within each cluster (given in pixels) of the same experiment used to provide the images in Figure S2. Slides that had at least 2 patches selected were analyzed with respect to the maximum distance between every patch pair and also the mean distance between pairs. These measurements confirm that DADA produces patch clusters that group similar visual characteristics but that are spread along the slide.

In opposition to the diversity of patches produced by the clustering system used by ENS DADA, the ENS method acquires the most uncertain images from the unlabelled pool. Figure S3 shows the 30 most uncertain images from the pool, which are selected by the ENS approach, and it is clear how they have very similar visual characteristics, as expected. This difference among DADA and other approaches is one of the key aspects of our solution, when uncertainties and diversity are combined in the selection strategy to accelerate model learning.

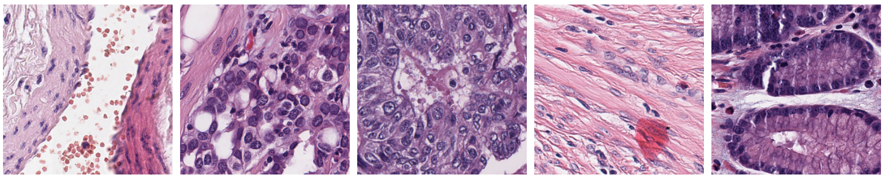

(a) Cluster #0.

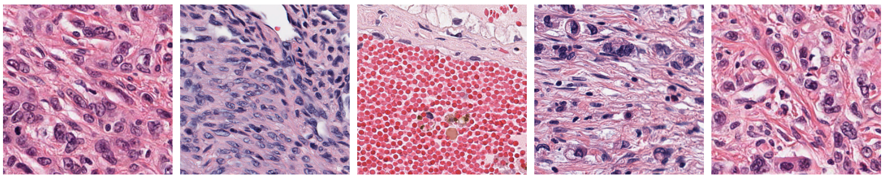

(b) Cluster #1.

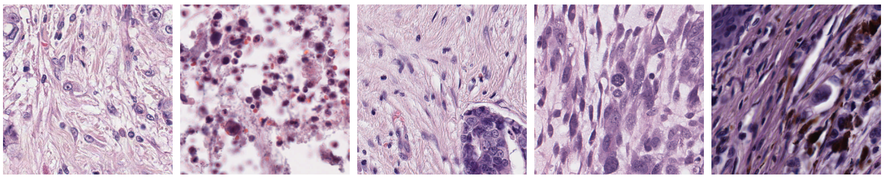

(c) Cluster #2.

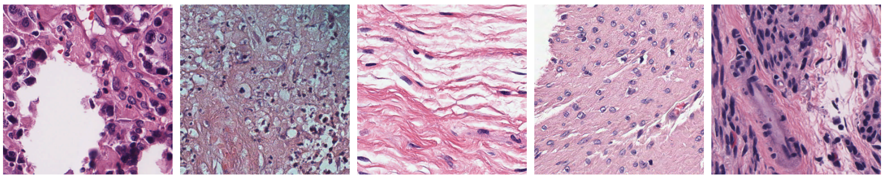

(d) Cluster #3.

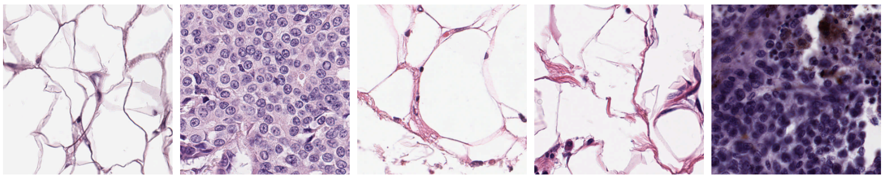

(e) Cluster #4.

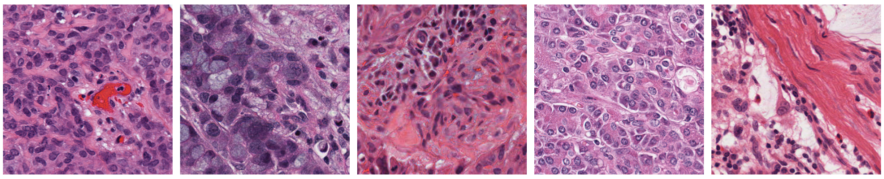

(f) Cluster #5.

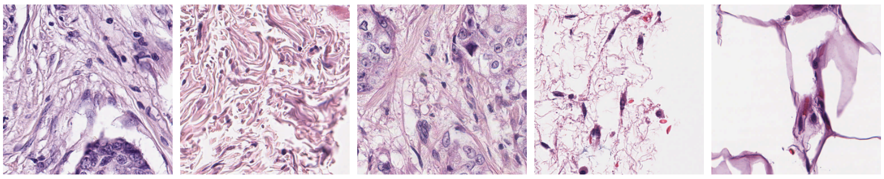

(g) Cluster #6.

Figure S2: Panels of patches in clusters 0 to 6, acquired with ENS DADA in the last acquisition of a 20 iteration experiment.

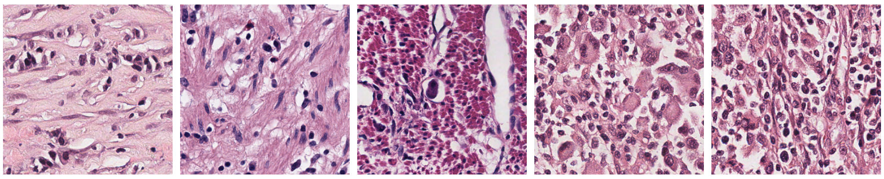

(h) Cluster #7.

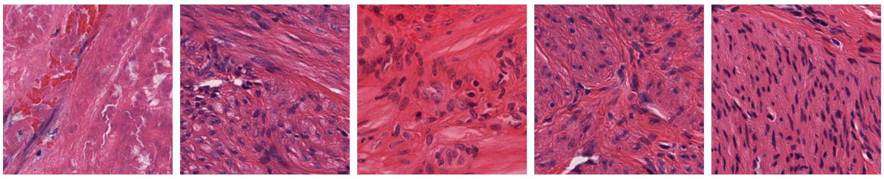

(i) Cluster #8.

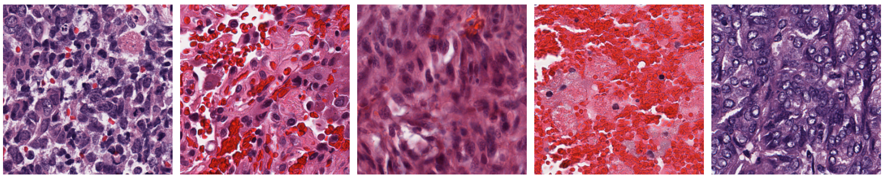

(j) Cluster #9.

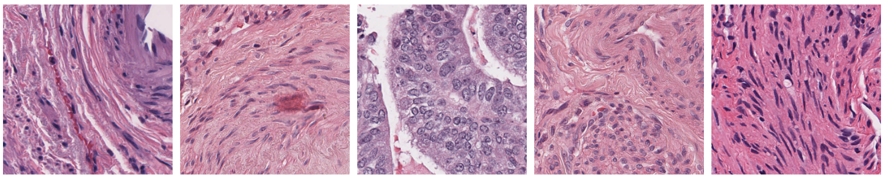

(k) Cluster #10.

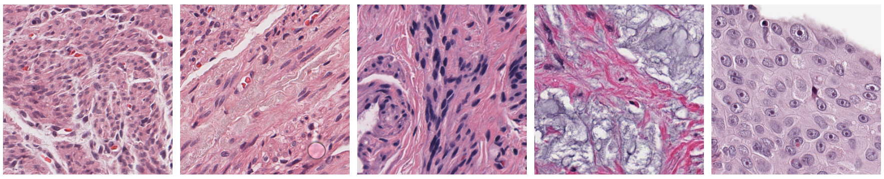

(l) Cluster #11.

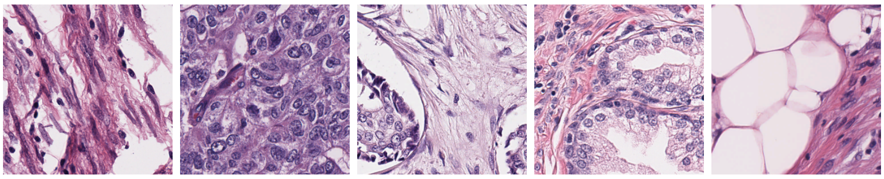

(m) Cluster #12.

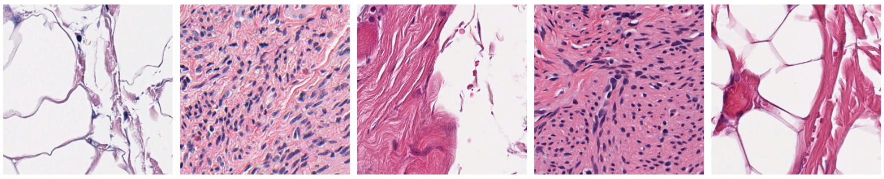

(n) Cluster #13.

Figure S2: Panels of patches in clusters 7 to 15, acquired with ENS DADA in the last acquisition of a 20 iteration experiment.

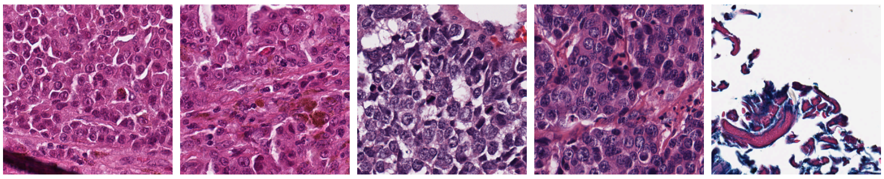

(o) Cluster #14.

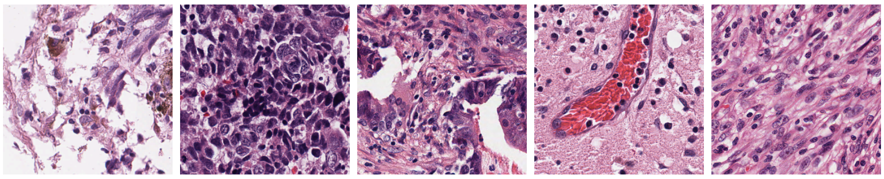

(p) Cluster #15.

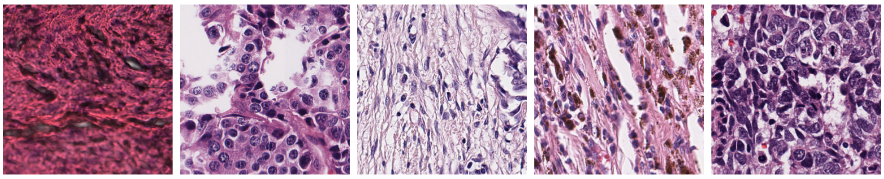

(q) Cluster #16.

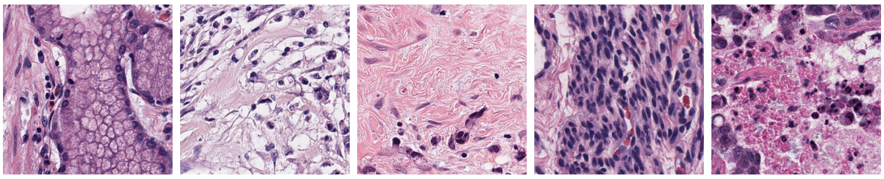

(r) Cluster #17.

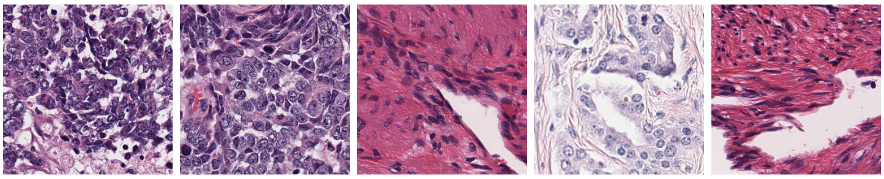

(s) Cluster #18.

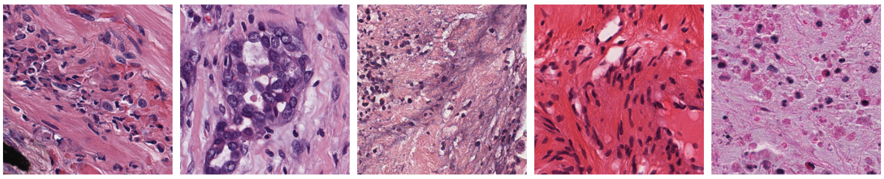

(t) Cluster #19.

Figure S2: Panels of patches acquired with ENS DADA in the last acquisition of a 20 iteration experiment.

Cluster #	# WSIs	Max Distance	Mean Distance
0	44	50,853.64	30,049.53
1	42	42,225.71	29,007.57
2	40	50,332.41	30,295.61
3	40	53,621.83	30,575.27
4	29	56,100.89	32,066.31
5	40	55,520.02	34,728.97
6	37	63,766.63	38,756.40
7	37	45,036.51	25,124.86
8	4	71,268.89	35,733.48
9	13	47,088.79	32,847.71
10	35	54,698.30	35,013.92
11	31	47,959.09	32,210.50
12	39	54,444.19	31,143.77
13	9	61,277.97	28,337.30
14	15	45,745.38	29,242.05
15	47	44,403.25	25,386.02
16	34	41,846.63	30,997.48
17	46	47,531.43	29,526.96
18	16	54,414.88	40,227.31
19	34	52,006.51	34,118.83

Table S8: Cluster setup for the last acquisition iteration of an ENS DADA experiment. Maximum and mean distances are given in pixels in the original slide and correspond to the cluster average.

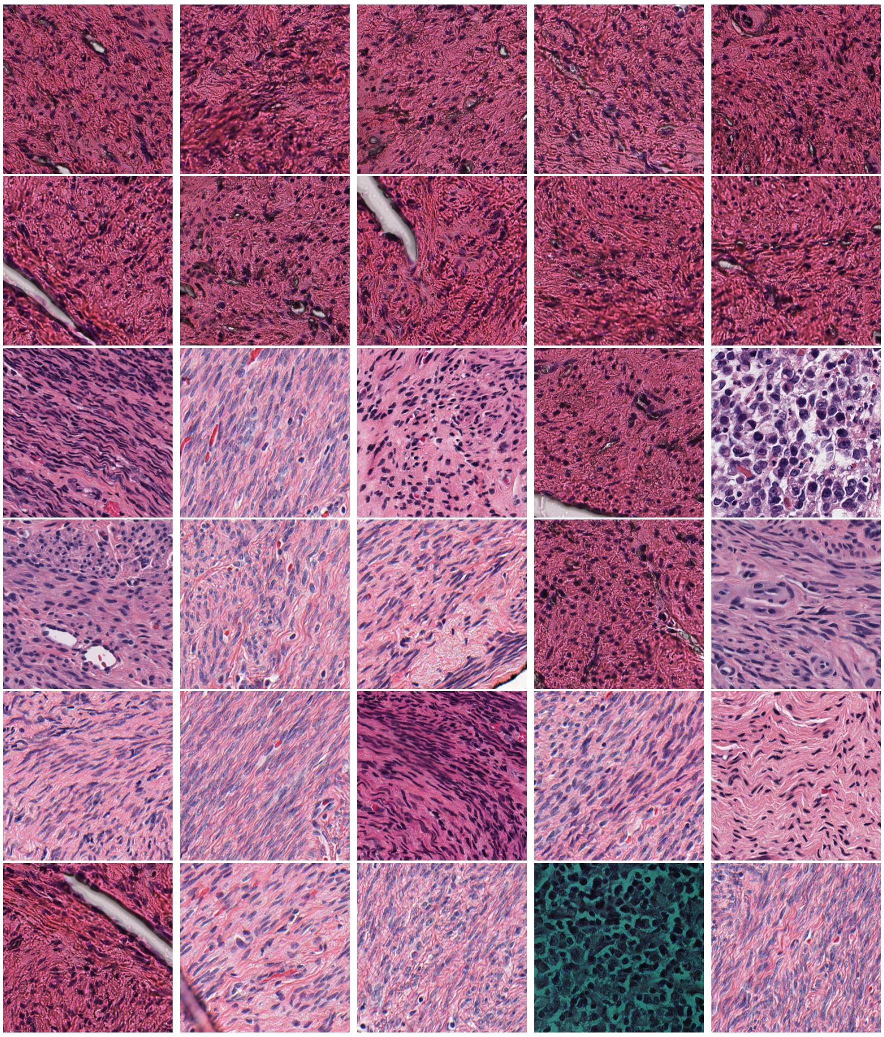

Figure S3: Panel of the 30 most uncertain patches, acquired with ENS method in the last acquisition of a 14 iteration experiment.

S4 Impact of ϕ to Other CNNs in AL

The ϕ parameter serves as a trade-off between execution cost and target network AUC. As presented in the main paper document, there are circumstances where higher selections of ϕ correspond to considerable reductions in execution time and only marginal to no loss in AUC, when using the Inception V4 network.

In this section, experiments conducted with other models, the Xception and ResNet50 V2, are presented and similar behavior was observed. A first observation is that these architectures have different classification performances, as demonstrated by Figure S4a, and have varying iteration execution times, as shown in Figure S4b.

When NAR is used with these models, iteration times are drastically cut as was observed with the Inception network, with only marginal differences in AUC level in comparison to the full model (Figures S5 and S6).

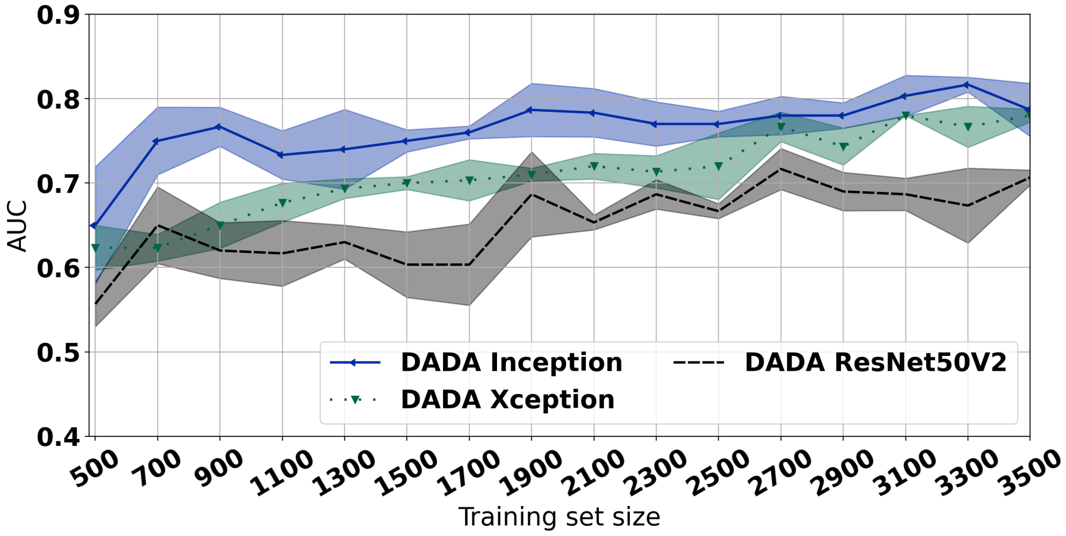

(a) AUC.

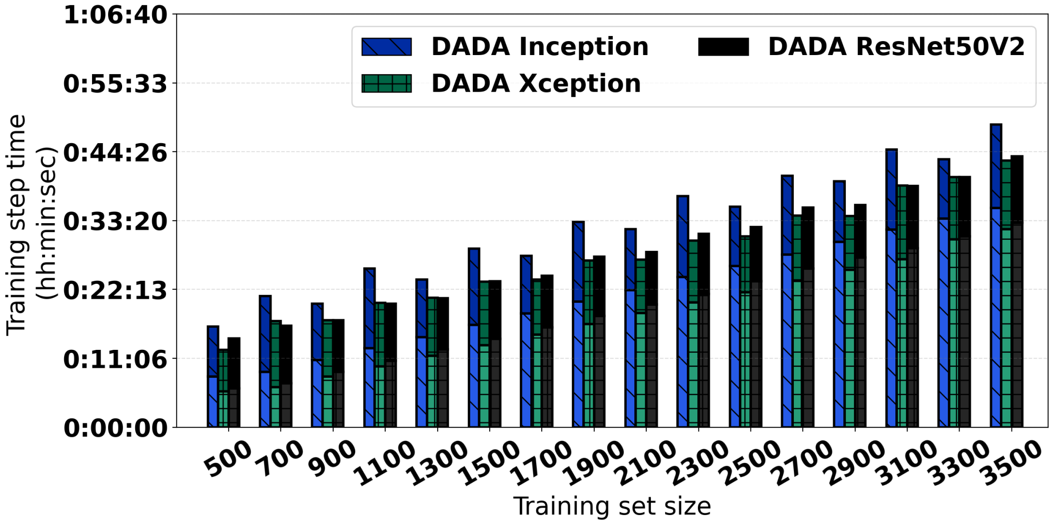

(b) Execution time.

Figure S4: MC DADA on multiple CNN architectures.

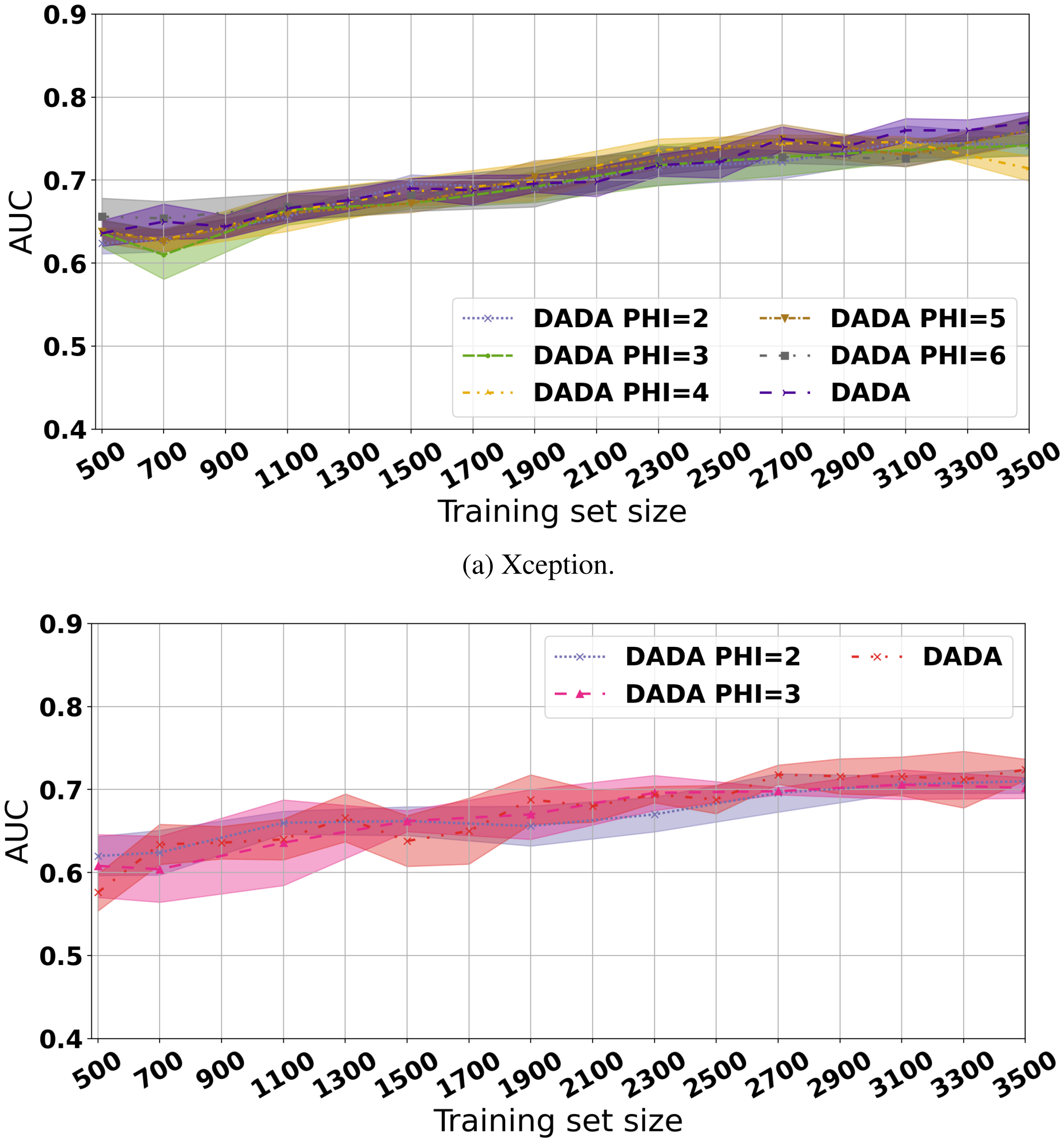

(a) Xception.

(b) ResNet50 V2.

Figure S5: Impact of NAR applied to the quality of the classification in the AL using MC DADA with Xception and ResNet50 V2 for multiple ϕ values.

S5 Varying TIL Datasets

The experiments conducted in the main paper use the same initial subpool and same 100k patches fixed pool (when applicable). If these sets are changed, a new TIL dataset is formed and used in the experiments. Although they represent the same application (TIL classification), these datasets have different patches and may pose other difficulties to the model.

Here we have also executed the AL with a new pool and used a new test set, which was also originated from the same slides listed in Table S4. The results are presented in Figure S7. As may be observed, a similar behavior was seen, when compared to the experiments displayed in main text. Both MC and Ensembles DADA approaches have close AUC levels and produce successive gains along time. This corroborates the first experiments that showed learning gains along training, with different difficulty levels presented by these datasets.

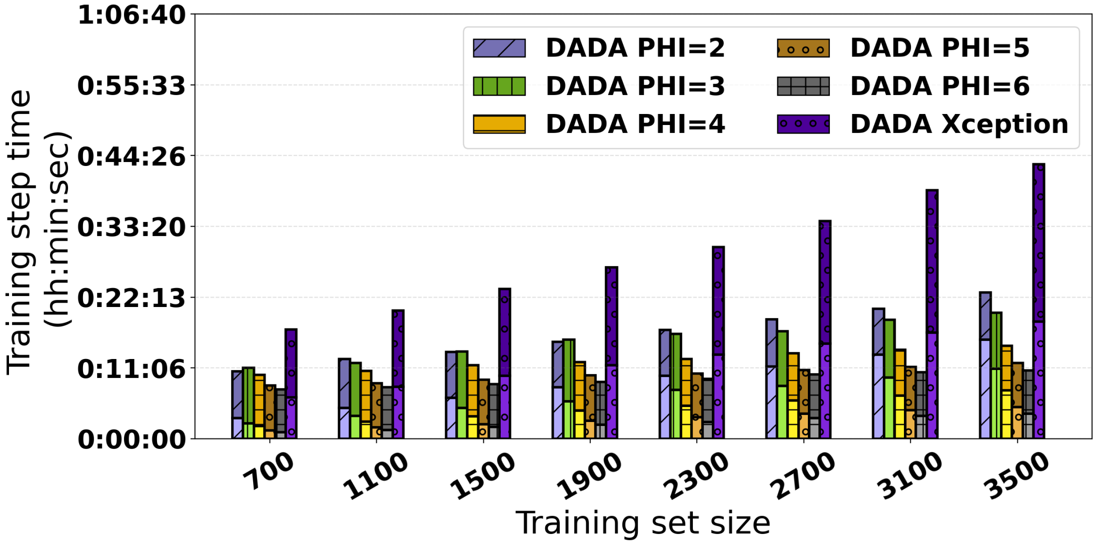

(a) Xception execution times.

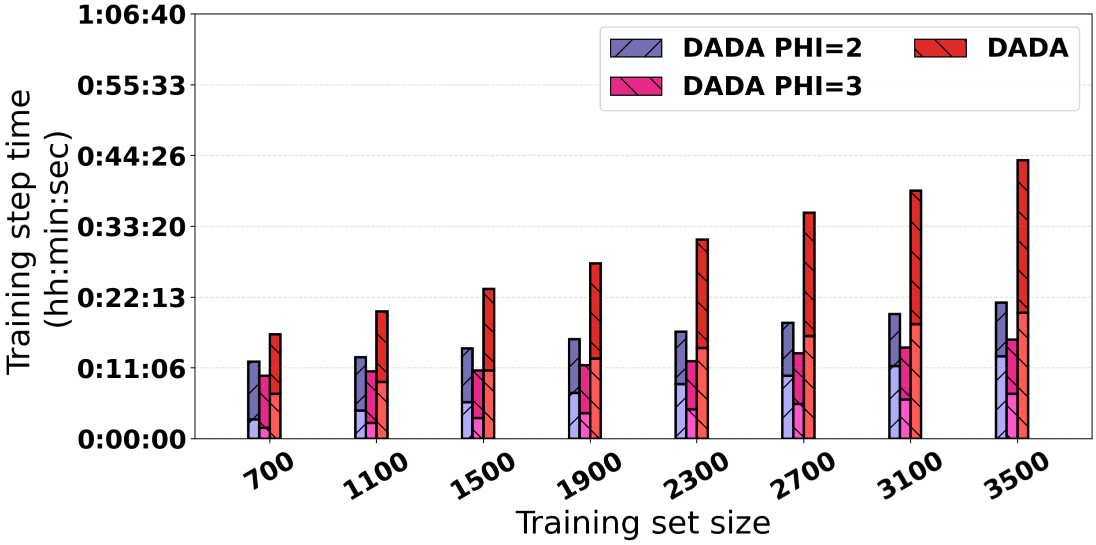

(b) ResNet50 V2 execution times.

Figure S6: Impact of NAR to execution time of AL with XCeption and ResNet50 V2 for multiple ϕ values.

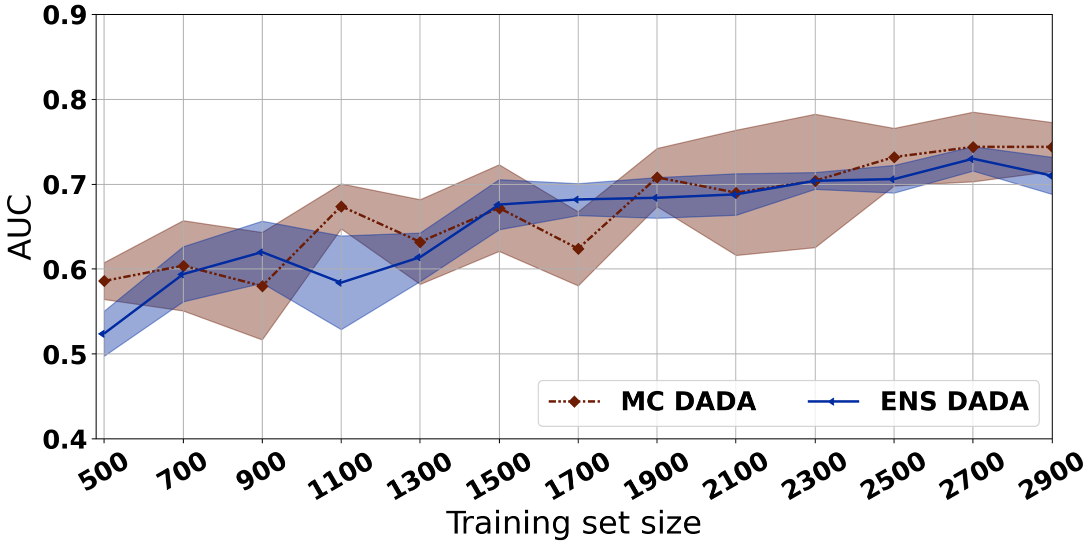

Figure S7: DADA evaluation with a different pool and test set of patches.

References

Meirelles, A. L., Kurc, T., Saltz, J., and Teodoro, G. (2022). Effective active learning in digital pathology: A case study in tumor infiltrating lymphocytes. *Computer Methods and Programs in Biomedicine*, **220**, 106828.

National Human Genome Research Institute (2017). The Cancer Genome Atlas. https://cancergenome.nih.gov/.

Saltz, J., Gupta, R., Hou, L., Kurc, T., Singh, P., Nguyen, V., Samaras, D., Shroyer, K. R., Zhao, T., Batiste, R., *et al.* (2018). Spatial organization and molecular correlation of tumor-infiltrating lymphocytes using deep learning on pathology images. *Cell reports*, **23**(1), 181.
